# Molecular Properties of *Flammulina velutipes* Polysaccharide–Whey Protein Isolate (WPI) Complexes via Noncovalent Interactions

**DOI:** 10.3390/foods10010001

**Published:** 2020-12-22

**Authors:** Jiaqi Shang, Minhe Liao, Ritian Jin, Xiangyu Teng, Hao Li, Yan Xu, Ligang Zhang, Ning Liu

**Affiliations:** 1 Key Laboratory of Dairy Science, Ministry of Education, Harbin 150030, China; ashang10@outlook.com (J.S.); minheliao@outlook.com (M.L.); ritianjin@outlook.com (R.J.); txy957180709@gmail.com (X.T.); bluesboylee@outlook.com (H.L.); yanxu1991@neau.edu.cn (Y.X.); 2 College of Food Science, Northeast Agricultural University, Harbin 150030, China

**Keywords:** *Flammulina velutipes*, protein–polysaccharide complexes, stability, bio-layer interferometry, in vitro digestibility, binding regions

## Abstract

Whey protein isolate (WPI) has a variety of nutritional benefits. The stability of WPI beverages has attracted a large amount of attention. In this study, *Flammulina velutipes* polysaccharides (FVPs) interacted with WPI to improve the stability via noncovalent interactions. Multiple light scattering studies showed that FVPs can improve the stability of WPI solutions, with results of radical scavenging activity assays demonstrating that the solutions of the complex had antioxidant activity. The addition of FVPs significantly altered the secondary structures of WPI, including its α-helix and random coil. The results of bio-layer interferometry (BLI) analysis indicated that FVPs interacted with the WPI, and the equilibrium dissociation constant (*K_D_*) was calculated as 1.736 × 10^−4^ M in this study. The in vitro digestibility studies showed that the FVPs protected WPI from pepsin digestion, increasing the satiety. Therefore, FVPs effectively interact with WPI through noncovalent interactions and improve the stability of WPI, with this method expected to be used in protein-enriched and functional beverages.

## 1. Introduction

Whey protein is a natural byproduct of cheese production and has high nutritional value. The molecular structure of whey protein has abundant branched-chain amino acids (BCAA; leucine, isoleucine, and valine), and its total amino acid composition is similar to that of skeletal muscles [1,2,3]. Whey protein isolate (WPI) is a class of whey protein that is often added to fitness, exercise nutrition, and weight management foods [4,5]. Many WPI-containing beverages are subjected to low pH (pH < 3.5), and the astringency is obvious at low pH [6]. The pH range with the best taste for WPI beverages at low pH is 4.0–6.0, as the isoelectric point (pI) of WPI is 5.1; however, making WPI stable near its pI is a problem.

It is now well recognized that the stability of proteins can be improved by conjunction with polysaccharides via covalent or noncovalent interactions [7,8,9]. Covalent interactions always occur through Maillard reactions, while noncovalent interactions result mainly from hydrophobic interactions, hydrogen binding, or electrostatic attraction under various conditions [10,11,12]. Maillard reactions in food may lead to the nutrients loss, and the contents of some proteins in these food are decreased or become non-digestible [13]. High temperature, time-consuming nature, and other parameters in the generation of food products may have harmful effects to health, such as mutagenicity and metabolic diseases [14]. When the pH of the reaction system is lower than the pI of protein, a strong electrostatic attraction occurs between the inversely charged protein and polysaccharide, thus forms a strong electrostatic complex (the pI is the pH value at which the surface of a molecule is uncharged) [15]. Therefore, the electrostatic attraction can make the protein and polysaccharide complexes keep the nutritional values without promoting detrimental health effects.

Previous studies have studied the interactions between polysaccharides and proteins. It has been reported that the presence of dextran sulfate (DS) decreased the turbidity of heated β-lactoglobulin (β-LG) and improved heat stability of β-LG [8]. However, changes in noncovalent interactions on structures of proteins in the composite system have yet to be resolved, and few studies have investigated the noncovalent interactions in the complex of WPI and bioactive polysaccharides. *Flammulina velutipes* belongs to Tricholomataceae and is widely eaten in China and Japan [16]. *Flammulina velutipes* polysaccharides (FVPs) are regarded as one of the most important substances in *Flammulina velutipes,* which have been reported to possess a wide spectrum of biological activities including antioxidant and hepatoprotective activities, the ability to prevent Hepatitis B Virus (HBV) infection, and the capacity to induce immunity by activating macrophages [17,18,19,20]. The molecular structure of polysaccharides is usually composed of many hydrophilic groups [21]. Hence, polysaccharides could have a beneficial impact on the stability of the WPI system.

The aim of this study was to investigate whether FVP could improve stability of WPI by occurring noncovalent interaction. In this study, FVPs were used as a stabilizer of the WPI system. The influence of adding FVP on particles properties and stability of WPI in aqueous solution was investigated. In addition, we investigated the antioxidant activity via the scavenging activity of three radicals. The changes of structure in WPI after noncovalent interaction with FVPs occurred were indicated. Then, we verified that there was an interaction between FVPs and WPI via the reaction kinetics. Finally, the digestibility and binding sites were assessed to understand the interaction between FVPs and WPI more stereoscopically. The results of this study should facilitate the development of new methods of WPI stabilizing with applications in the food industry.

## 2. Materials and Methods

### 2.1. Materials

The *Flammulina velutipes* used in this study originated from the Chinese mainland and were purchased from a B.U.T. Mart in Harbin. WPI was from Davisco Foods International (Le Sueur, MN, USA). Nitro blue tetrazolium (NBT) and phenazine methosulfate (PMS) were from Sigma-Aldrich (St. Louis, MO, USA). EZ-Link NHS-PEG_12_-Biotin (21312) and trypsin (MS-grade, 90057, ≥15,000 u/mg) were from Thermo Scientific (Waltham, MA, USA). Other chemicals were purchased from the Aladdin reagent official website.

### 2.2. Extraction of FVPs

The FVPs extraction was performed according to a previously described method with vacuum concentration [22,23]. In brief, *Flammulina velutipes* powder was extracted with distilled water at 85 °C for 2 h. Then, the extracts were concentrated at 50 °C. After concentration, the extracts were centrifuged at 4000 rpm for 30 min. Subsequently, the supernatant was collected and precipitated using 4 volumes of absolute ethanol. The resulting precipitate was collected and freeze-dried (crude polysaccharide). Proteins were removed from the sediment using the Sevag method. Briefly, Sevag reagent contained chloroform and n-butanol solvents at a volume ratio of 1:5. A total of 3 mg/mL crude polysaccharide solution was added with the Sevag reagent at a volume ratio of 1:3. The well-mixed solution was stirred for 20 min and stood undisturbed to form the phase-separation. The supernatant was collected, and 3 steps were repeated 5 times. Furthermore, the samples were decolorized with activated carbon after deproteinization. Finally, the FVPs were filtered by air pump filtration and dried on a freeze dryer (Marin Christ Alpha 2–4 LSCplus, Osterode, Germany).

### 2.3. Preparation of FVP–WPI Complex Solutions

The FVP–WPI complex solutions were prepared according a previously described method with different ratios [24]. The solutions of FVP–WPI complex with final concentrations of 0.4% (*w*/*w*) FVPs and 3–7% (*w*/*w*) WPI were obtained using a 0.8% FVP stock solution and 6–14% WPI stock solutions, which were stored at 4 °C overnight. FVP and WPI stock solutions were mixed at the weight ratio of 1:1. The pH of solutions was adjusted to 4.5 with 1 M NaOH and 1 M HCl, after which 20 mL of each solution was transferred to a flat-bottomed cylindrical glass measurement cell with a black cap for stability analysis.

### 2.4. Particle Size and Zeta Potential

The apparent size and zeta potential of the samples were analyzed by Dynamic Light Scattering (Malvern Zetasizer Nano ZS, Worcestershire, UK) using a previously described method [25]. Distilled water was used to dilute the sample solutions at a ratio of 1:200, and the pH of solutions was adjusted to 4.5 with 1 M NaOH and 1 M HCl. Samples were well mixed before measurement. Each sample was measured 3 times, and the results were averaged. The cell was maintained at room temperature.

### 2.5. Stability Monitoring

Stability was monitored using multiple light scattering (Formulaction TURBISCAN TOWER, Toulouse, France) by using a described scheme of the equipment [26]. Multiple light scattering is used to comprehensively characterize the stability characteristics of high-concentration dispersion systems. The samples were scanned from the bottom to the top in order to monitor the optical properties of the dispersion along the height in the cell. The sample was stored at 4 °C and monitored after 0, 5, 10, and 15 d. Each monitoring duration was 30 min, and scanning was performed once in every 110 s for 17 times totally. The stability of the samples was deduced from the back scattering (BS) data.

### 2.6. Antioxidant Activity

#### 2.6.1. ABTS Radical Scavenging Activity

The 2,2′-azinobis (3-ethylbenzothiazoline-6-sulphonic acid) (ABTS) radical scavenging activities of the samples were determined according to a previously described method with 7 mM ABTS [27]. In brief, an adequate volume of 7 mM ABTS was added to the same volume of 2.45 mM potassium persulfate solution, after which the mixture was incubated in the dark for 12 h at room temperature before being diluted with PBS (phosphate-buffered saline, 0.01 M, pH 7.4) to attain an absorbance of the working solution of 0.70 ± 0.02 at 734 nm. Then, 1.0 mL sample was mixed with 4.0 mL of the ABTS working solution and vortexed. The absorbance of the final mixture was read at 734 nm using the microplate reader (Molecular Devices M2e, San Francisco, CA, USA), and the ABTS radical scavenging activity was calculated using Equation (1).

ABTS radical scavenging activity (%) = [1 − (A_1_ − A_2_)/A_0_] × 100 (1)

where A_0_ is the Abs_734_ of the mixture without sample, A_1_ is the Abs_734_ of the sample, and A_2_ is the Abs_734_ of the sample without ABTS radical solution.

#### 2.6.2. Hydroxyl Radical Scavenging Activity

The hydroxyl radical scavenging activities of the samples were determined by a reported method [28]. In brief, 0.5 mL of 9 mM ferrous sulfate solution was mixed with 1.0 mL of 8.8 mM hydrogen peroxide solution to perform the Fenton reaction. Then, 1 mL sample solution was added to the Fenton reaction mixture. Finally, 0.1 mL 9 mM salicylic acid (dissolved in ethanol) was added to the mixture and vortexed, after which the mixture was incubated at 37 °C for 1 h. The absorbance of the final mixture was read at 510 nm on a microplate reader, and the hydroxyl radical scavenging activity was calculated using Equation (2).

Hydroxyl radical scavenging activity (%) = [1 − (A_1_ − A_2_)/A_0_] × 100 (2)

where A_0_ is the Abs_510_ of the control (without sample), A_1_ is the Abs_510_ of the sample with salicylic acid, and A_2_ is the Abs_510_ of the sample without salicylic acid.

#### 2.6.3. Superoxide Anion Radical Scavenging Activity

The superoxide anion radical scavenging activities of the samples were determined using a reported method [29]. Briefly, 1 mL sample solution was mixed with 1 mL 300 μM NBT, after which 1 mL 936 μM NADH (nicotinamide adenine dinucleotide) was added to the mixture. Finally, 1 mL 120 μM PMS was added to the reaction mixture before it was incubated at 25 °C for 5 min. The absorbance of the final mixture was read at 560 nm, and the superoxide anion radical scavenging activity was calculated using Equation (3).

Superoxide anion scavenging activity (%) = (A_0_ − A_1_)/A_0_ × 100 (3)

where A_0_ is the Abs_560_ of the control (without sample), and A_1_ is the Abs_560_ of the sample.

### 2.7. FTIR

FTIR analysis was performed using an FTIR spectrometer (NICOLET, Madison, USA) according to the method reported by Yan et al. [29]. The lyophilized powders of FVPs, WPI, and FVP–WPI were grounded with KBr powder and then pressed into pellets for FTIR measurements. FTIR spectra were recorded at a wavelength range of 400–4000 cm^−1^.

### 2.8. Raman Spectroscopy

Raman spectra of FVPs, WPI, and 0.4% FVP–5% WPI were conducted using a Raman spectrometer (HORIBA Evilution, France) equipped with a microscope and a 532 nm near-infrared diode laser according to a reported method with different detection ranges [30]. Each sample was deposited onto a microscope slide and freeze-dried prior to Raman measurement. The Raman spectra were recorded at room temperature with a detection range from 400–2800 cm^−1^. Spectral data were collected using LabSpec6 and were baseline corrected and normalized according to the protein phenylalanine peak at 1003 ± 1 cm^−1^.

### 2.9. XRD

The XRD spectra were performed according to Liu et al., with different ranges of scan [31]. The XRD spectra were performed using an X-ray diffractometer (Fangyuan 2700, Dandong, China) at 40 mA and 40 kV, where the target material was Cu. The scattering angle (2θ) was scanned over the range of 5–80° at a scanning step of 0.05°. Spectral data were collected and further analyzed using MDJ Jade 6.0.

### 2.10. Reaction Kinetics

Recently, bio-layer interferometry (BLI) was developed to assess molecular interactions [32,33]. The reaction kinetics were determined using an unlabeled molecular interaction analysis system (Pall ForteBio Octet RED96e, Fremont, CA, USA). The biotinylation of WPI was performed using EZ-Link NHS-PEG_12_-Biotin according to the specification, where 1 mL 1 mg/mL WPI stock solution was mixed with 3 μL of 10 mM EZ-Link NHS-PEG_12_-Biotin mother liquor and vortexed. The reaction mixtures were incubated at room temperature for 30 min, after which the free biotin was removed using a Zeba desalination centrifugal column (Thermo Scientific). Then, 600 μL biotinylated WPI was added to a preprocessed desalination centrifugal column and centrifuged at 1000× *g* for 2 min to collect the target protein. The protein concentration was determined using a BCA Protein Assay kit according to the manufactures’ instructions (Meilunbio MA0082, Dalian, China). The biotinylated WPI was diluted to 20 μg/mL in PBS (0.01 M). The test was conducted using a previously reported method without running buffer (containing BSA) [34,35]. In each assay, the streptavidin (SA) biosensor was balanced with PBS for 60 s before being loaded with biotinylated WPI for 120 s to achieve a loading signal between 2.0 and 2.1 nm, which was followed by a balancing step in PBST (0.01 M PBS + 0.02% Tween) for 120 s. Subsequently, association of WPI with FVPs at concentrations of 31.25, 62.5, 125, 250, 500, and 1000 μg/mL were performed for 240 s. Finally, the dissociation was monitored in PBST for 120 s.

### 2.11. Protein Digestion

In vitro pepsin digestion in this study was performed according to methods reported by Akl et al. [36]. Simulated gastric fluid (SGF) was composed of 0.2% NaCl, 0.32% pepsin (≥250 u/mg), and 0.7% HCl (36.5%) according to the United States Pharmacopeial Convention. After diluting 7 mL of HCl in an appropriate volume of water, we added 3.2 g of pepsin and 2 g NaCl to the HCl solution. Then, the mixture was diluted to 1000 mL. SGF was prepared immediately before the digestion. First, 10 mL SGF was incubated with continuous shaking at 95 rpm for 5 min at 37 °C in a temperature-controlled incubator. Then, 10 mL WPI or 0.4% FVP–5% WPI sample solution was added into the reaction system. The pH of the solution was adjusted to 7.0 with 1 M NaOH after reacting for 10 or 120 min, respectively.

Trypsin digestion was carried out according to methods promoted by Zhang et al. with minor modifications [37]. First, the pepsin-digested samples were diluted to 10 μg/μL. Then, 20 μL diluted samples were mixed with 40 μL of protein denaturation solution (6 M GdmCl, 100 mM Tris, 10 mM Tris-(2-cyanoethyl) phophine, 40 mM Bis (2,4-pentanedionato) calcium, pH 8.5). After being heated at 95 °C for 5 min, the solution was transferred to an ultrafiltration tube (molecular weight cutoff: 10 kDa; Millipore) after cooling and centrifuged at 14,000 rpm for 15 min. The peptide fragments greater than 10 kDa were washed twice with 50 mM ammonium bicarbonate solution. Then, trypsin was added to the solution with a final ratio 50:1 (*w*/*w*, protein/enzyme), and the well-mixed solution was reacted at 37 °C for 16 h. Finally, the mixture in the ultrafiltration tube was centrifuged at 14,000 rpm for 15 min, and the effluents were used for proteomic analysis. Triple replicates were performed for each sample.

### 2.12. Proteomic Analysis

A nano-liquid phase system (Thermo Scientific Easy nLC1200, Waltham, MA, USA) was used to isolate the peptides according to the manufacturer’s instructions. Solvent A was water (0.1% formic acid), and solvent B was 80% acetonitrile (0.1% formic acid). The chromatographic column was equilibrated using mobile phase A, and the samples were loaded by an automatic sampler and separated at a flow rate of 300 nL/min using gradient conditions described in Table 1. The samples were analyzed by mass spectrometry (Thermo Scientific Q-EXACTIVE PLUS, Waltham, MA, USA), with survey scans of peptide precursors performed from 350 to 1800 *m*/*z* at 200 *m*/*z*. After each survey scan, the AGC target for MS/MS was set to 3 × 10^6^, and the maximum injection time was limited to 50 ms.

All data were obtained in *. raw format, and the data files were analyzed against transforming growth factor beta-1 proprotein (P18341), transforming growth factor beta-2 proprotein (P21214), α-lactalbumin (P00711), β-lactoglobulin, (P02754), serum albumin (P02769), lactoperoxidase (P80025), and lactotransferrin (P24627), downloaded from the UniProt database. After the analysis of the quantification data in MaxQuant to obtain the “Peptides.txt” file, intensities of the “Peptides.txt” data were log_2_ transformed. Then, a *t*-test was performed to analyze the significance using Perseus.

### 2.13. Statistical Analysis

Each experiment was performed 3 times. Results were presented as mean value ± standard deviation (SD). Statistical analysis of the results was performed by one-way analysis of variance (ANOVA) and Duncan’s test using the SPSS software. Values of *p* < 0.05 were considered to be statistically significant.

## 3. Results

### 3.1. Characterization of WPI and FVP–WPI Particles

**Figure 1:**
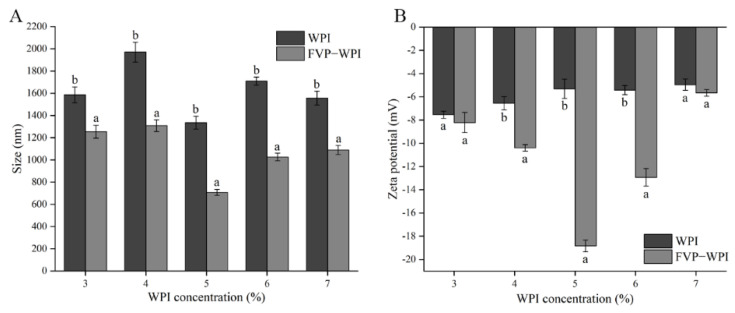
Characterization of whey protein isolate (WPI) and *Flammulina velutipes* polysaccharide (FVP)–WPI particles at pH 4.5. (**A**) Particle size; (**B**) zeta potential. Different letters in the figure represent significant differences in different samples of the same concentration (*p* < 0.05).

The formation of FVP–WPI complex changed the properties of WPI particles, which can be assessed by the measurement of the particle size and zeta potential. The particle size of WPI was 189.68 nm at pH 7.0, and when the pH was adjusted to 4.5, the particle size of WPI increased to 1586.45 ± 70.67 nm (3% WPI), 1970.33 ± 88.85 nm (4% WPI), 1336 ± 58.05 nm (5% WPI), 1709.33 ± 36.30 nm (6% WPI), and 1557.00 ± 63.27 nm (7% WPI). This phenomenon may be attributed to the pH near the pI of WPI. The apparent sizes of the FVP–WPI complexes and WPI at pH 4.5 are presented in Figure 1A, which showed that the particle sizes for the FVP–WPI complexes were smaller than those measured in solutions with WPI alone. Furthermore, smallest particle sizes were observed for WPI and FVP–WPI complexes obtained with a 5% WPI concentration, for which particle sizes in the solutions of WPI and solutions of FVP–WPI complex were approximately 1336 ± 58.05 and 707.13 ± 26.17 nm, respectively. Zhao et al. reported that the addition of soybean soluble polysaccharides and beet pectin to lactoferrin emulsions increase the stability during storage, and emulsions containing protein only show change in size during the storage of 2 weeks (from 209 nm to around 1000 nm) [38]. This result may be due to the electrostatic interactions of polysaccharides on the protein surface that inhibit protein aggregation.

Zeta potential measurements are used to evaluate the stability of a dispersion system; the high absolute value of zeta potential indicates that there is a greater electrostatic repulsion between two molecules and the system is more stable. The stability of the system is affected by particle size and particle surface charge in the dispersed system [39]. The zeta potential of the investigated polysaccharide was −12.53 ± 0.51 mV. The zeta potential values of WPI and FVP–WPI complexes are shown in Figure 1B. These results showed that the noncovalent interactions between FVPs and WPI led to a decrease in the zeta potential of the solutions of the FVP–WPI complex. The zeta potentials of the WPI solutions ranged from −7.54 ± 0.31 mV at 3% WPI to −4.95 ± 0.49 mV at 7% WPI. In contrast, the zeta potential values of FVP–WPI complexes showed a low valley, decreasing from −8.22 ± 0.86 to −18.83 ± 0.50 mV as the WPI concentration increased from 3 to 5% since the charged area of the WPI surface was masked by FVPs as the complex formed. Interestingly, it has been previously reported that the zeta potential of protein decreases when interacting with polysaccharides [40,41]. Therefore, when the concentration of WPI increased, the surface potential of FVP–WPI decreased, and the number of FVP–WPI composite particles increased. However, the potential increased when the WPI concentration was 6 or 7%—this may be attributed to the surface of WPI that could not be covered by FVPs.

It could be observed in Figure 1 that when the concentration of WPI was 5%, the particle size and potential of FVP–WPI were the lowest. With the increase of WPI concentration, the particle size and potential of FVP–WPI increased. We confirmed that the best ratio of noncovalent interactions between FVPs and WPI was 1:12.5.

### 3.2. Turbiscan Measurements of Stability

**Figure 2:**
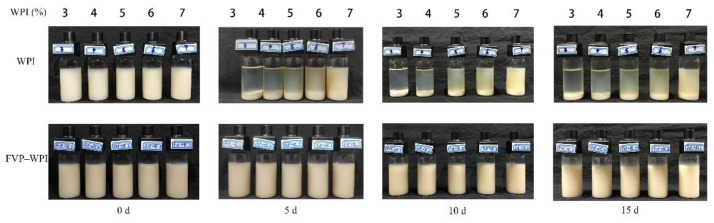
Images of WPI and FVP–WPI solutions over 15 d of storage.

Images of the WPI and FVP–WPI solutions stored from 0 to 15 d at 4 °C are presented in Figure 2. When the pH was adjusted to 4.5, precipitates were observed at the bottom of the cells for the WPI solutions at all assayed concentrations. The supernatant became more and more turbid with the increase of WPI concentrations. However, the solutions of FVP–WPI complex displayed better dispersal than the WPI solutions. No phase separation occurred in the FVP–WPI solutions after being stored for 15 d.

**Figure 3:**
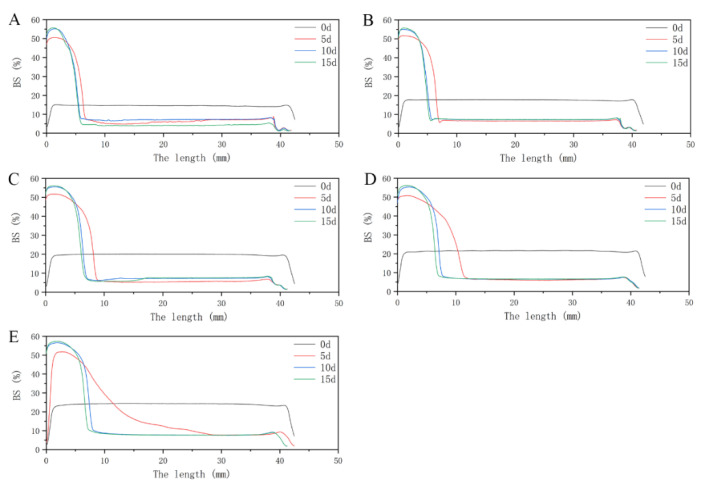
Back scattering (BS) of WPI solutions over storage time under static conditions. (**A**) 3% WPI solution; (**B**) 4% WPI solution; (**C**) 5% WPI solution; (**D**) 6% WPI solution; (**E**) 7% WPI solution.

**Figure 4:**
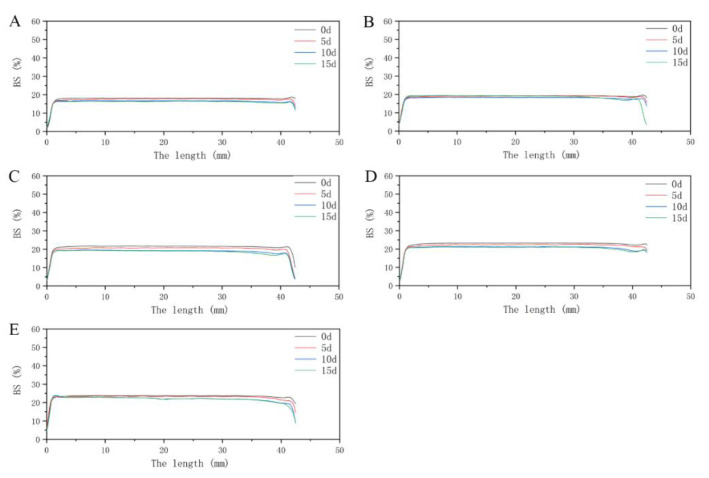
Back scattering (BS) of FVP–WPI solutions over storage time under static conditions. (**A**) 0.4% FVP–3% WPI solution; (**B**) 0.4% FVP–4% WPI solution; (**C**) 0.4% FVP–5% WPI solution; (**D**) 0.4% FVP–6% WPI solution; (**E**) 0.4% FVP–7% WPI solution.

Turbiscan is commonly used to predict and monitor the stability of systems [42]. Figure 3 and Figure 4 show the BS of WPI and FVP–WPI solutions obtained at different times for the samples formulated from 3% WPI to 7% WPI via Turbiscan. All samples were measured on days 0, 5, 10, and 15. During this period, the FVP–WPI solutions showed better stabilities than the WPI solutions, and the destabilization of WPI solutions were caused by sedimentation. Sedimentation was evidenced by the increasing BS at the bottom zone (0–10 mm)—the range of length increased with the increasing of WPI concentrations. For all the solutions of FVP–WPI complex, the BS remained with no change over 15 d of storage, showing that no sedimentation occurred. In order to further verify the stability of FVP–WPI solutions, we carried out an accelerated stability test for 60 days according to a previously described method [43]. Images and BS of the FVP–WPI solutions stored from 0 to 60 d at 40 °C are presented in Figure S1. As a result, there was no phase separation in the heated FVP–WPI solutions during storage. The study of Yin et al. shows that soy polysaccharide and soy protein could form dispersible complexes at pH 3.25 in aqueous solution via electrostatic interactions [44]. These observations were consistent with the particle size and zeta potential analyses in Section 3.1, which indicated that the addition of FVPs can essentially maintain the stability of WPI at pH 4.5. The FVPs and WPI had opposite charges at pH 4.5, which caused the electrostatic attractions and drove the formation of complex through noncovalent interactions. We discovered that solution of FVP–WPI complex had higher storage stability than the WPI solutions.

### 3.3. Antioxidant Activities

**Figure 5:**
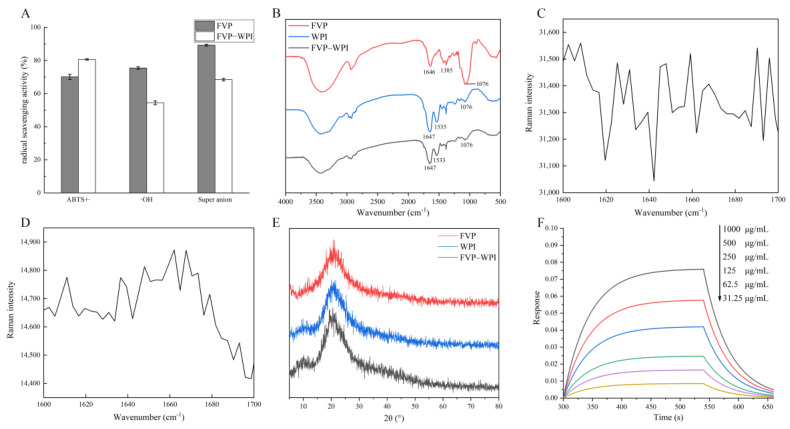
Antioxidant activities and structures of FVPs, WPI, and FVP–WPI. (**A**) The antioxidant activities of FVPs and FVP–WPI, (**B**) the FTIR spectra of the FVPs, WPI, and FVP–WPI complex, (**C**,**D**) the amide Ι bands of WPI and FVP–WPI in Raman spectra, (**E**) the XRD spectra of the WPI, FVPs, and FVP–WPI, (**F**) the fitting curves for association and dissociation of FVPs and WPI.

The antioxidant activities of 0.4% FVPs and 0.4% FVP–5% WPI were assessed using three radicals, including ABTS, hydroxyl, and superoxide anion radicals (Figure 5A). FVP–WPI complexes displayed better ABTS radical scavenging activity than the FVPs, whereas the FVPs exhibited better antioxidant activity than the FVP–WPI solution with respect to its hydroxyl and superoxide anion radical scavenging activities. The difference of hydroxyl radical scavenging activities between FVP and FVP–WPI may result from the uronic acid in FVP, which was masked by WPI during interaction with WPI. Some studies reported that the content of uronic acid in polysaccharides is related to their radical scavenging activity [45,46]. The result of superoxide anion radical scavenging activities may result from the change in conformation; the result in Section 3.5 showed that the conformation of FVPs shifted when they interacted with WPI. The study of Zhang et al. shows that the biological activities of lentinan are correlated with the conformation of the molecule, and the biological activity decreases when the conformation is destroyed [46,47]. This will be of importance in designing a product that is stabilized by polysaccharide–protein complexes with objectives of delivering nutrients with regard to health benefits simultaneously.

### 3.4. FTIR and Raman Spectroscopy

The FTIR spectra of the freeze-dried FVPs, WPI, and 0.4% FVP–5% WPI complex powders are presented in Figure 5B. In previous research, the strong band in the FVP spectrum at approximately 1076 cm^−1^ was ascribed to pyran-type structure, the absorption peak at approximately 1385 cm^−1^ was due to C–H deformation vibration, and the strong peak at approximately 1647 cm^−1^ was associated with C=O asymmetrical stretching vibrations [48,49]. In a previous study, the amide I and II peaks were related to the secondary structure, for instance, α-helix and β-sheet [50]. The amide I peak represented the C=O stretching vibrations, and the amide II peak was due to the N=H bending and C=N stretching vibrations. Our results showed that the amide I peak of WPI was observed at 1647 cm^−1^, and the amide II peak was observed at 1535 cm^−1^, with one band detected at 1076 cm^−1^. In addition, the amide I peak of the FVP–WPI spectrum was at 1647 cm^−1^, the amide II peak shifted to 1533 cm^−1^ compared with that observed in WPI, and a band at 1076 cm^−1^ was also observed. These results revealed that addition of FVPs had no clear effects on the positions of amide I and II peaks of WPI. Furthermore, we estimated the changes of protein secondary structure quantitatively through Raman spectra.

Changes of the position and intensity of the spectral peaks in Raman spectra reflect changes in protein structures [30]. Conformations of the primary protein chain were determined by the characteristic peak of amide Ι bands (1600–1700 cm^−1^), and the Raman spectra of WPI and 0.4% FVP–5% WPI are shown in Figure 5C,D. Fitting of the amide I band was performed typically to estimate protein secondary structure quantitatively, and the percentages of α-helix, β-sheet, β-turn, random coil, and amino acid side chains were calculated by the relative contributions of respective components. The frequencies and corresponding percentages of each secondary structure for the two samples obtained by performing fitting calculations are shown in Table 2. After noncovalent interactions, the α-helix, β-turn, and random coil contents were found to be higher in the FVP–WPI complex than in WPI (*p* < 0.05). Therefore, formation of the FVP–WPI complexes appeared to change the spatial secondary structure of WPI and promote the formation of α-helices, β-turns, and random coils in the FVP–WPI complexes. In our work, the variation trends of percentages of each secondary structure were different from those reported by Zhang et al., which may have been caused by diverse interactions or diverse polysaccharides [51]. We found the secondary structures of WPI were changed significantly after interacting with FVPs, including α-helix, β-turn, and random coil.

### 3.5. XRD Analysis

XRD analyses were performed to obtain crystal structures. The XRD spectra of the freeze-dried FVPs, WPI, and 0.4% FVP–5% WPI complex powders are presented in Figure 5E. Two peaks were observed at the reflections of approximately 10° and 21° in the WPI and FVP–WPI samples, while only one peak appeared in the spectrum of FVPs at a reflection of approximately 21°. The crystallinity of FVP–WPI was lower than FVPs or WPI, as there was noncovalent interactions with FVPs, indicating that the crystal became more amorphous, which was similar to the work of Xu et al. [52]. This result was due to the FVPs entering the crystalline structure of WPI, which resulted in the amorphous structure of the FVP–WPI complex.

### 3.6. Assessment of the FVP–WPI Interaction by BLI

The kinetics assay was performed using five sections, including two baselines (PBS and PBST), a loading of ligand (WPI), an association of the analyte (FVPs), and a dissociation step in PBST. We analyzed association and dissociation mainly as a result of the three sections (two baselines and a loading of ligand) in different concentrations assays, which were found to be consistent. The fitting curves for association and dissociation are shown in Figure 5F, and the *R^2^* value of the fitting curves was 0.99. In the gradient test, the FVPs exhibited a gradient interaction with the receptor protein WPI, and the equilibrium dissociation constant (*K_D_*) between WPI and FVPs was calculated as 1.736 × 10^−4^ M, suggesting that there was an interaction that occurred between FVPs and WPI. Similarly, Wallner recently showed in protein/liposome binding interactions based on the BLI that liposome formed stable complexes with the protein, wherein the *K_D_* between protein and liposome was calculated as 5.166 × 10^−5^ M–1.845 × 10^−4^ M, which was different from our study [53]. This difference may be due to bond strength of materials. We believe that BLI can be used to measure the kinetics in interactions between polysaccharides and proteins.

### 3.7. In Vitro Digestibility

**Figure 6:**
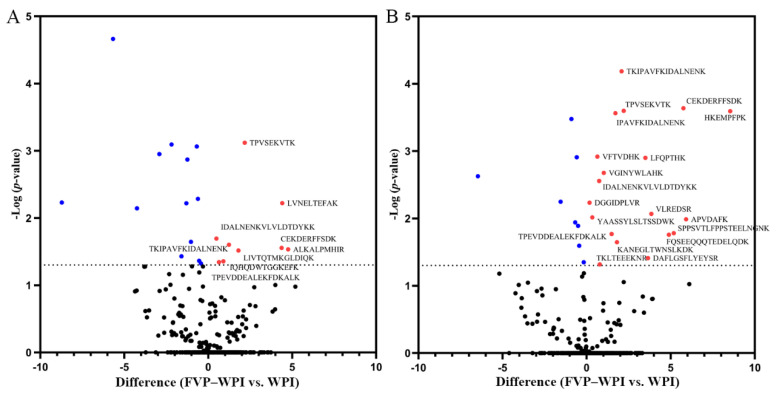
The volcano plots of the change in peptide abundance between FVP–WPI and WPI (control). (**A**) Differences of peptides digested for 10 min. (**B**) Differences of peptides digested for 120 min. Significant changes (*p*-value < 0.05) appear in the upper left (significant decrease) and upper right quadrants (significant increase) of the plot. The color is used to distinguish whether the sequences are different. Red circles represent significant increase and blue circles represent significant decrease.

A double digestion process in the proteomics analysis was used to produce peptides, after which the protein undergoing transformations at the peptide level were determined. The volcano plots were used to show the different peptides between FVP–WPI and WPI (the essence of a volcano plot is a scatter plot). The *y*-axis represented −log_10_ (*p*-value). The higher the *y*-coordinate value, the smaller the *p*-value, that is, the more significant. The *x*-axis represented difference (FVP–WPI vs. WPI). The positive *x*-coordinate value represented the fact that the peptide abundance of FVP–WPI was higher than the peptide abundance of WPI. The negative *x*-coordinate value represented the fact that the peptide abundance of FVP–WPI was smaller than the peptide abundance of WPI. Volcano plots of the 0.4% FVP–5% WPI complex compared with WPI are shown in Figure 6, where limited digestion by pepsin (non-specific protease) was performed (10 or 120 min), followed by complete proteolysis using trypsin (specific protease). After decompositions via pepsin and trypsin digestion, three types of peptides were generated, including peptides both having lysine (Lys, K) and arginine (Arg, R) terminals, peptides that have K or R terminals, and other peptides (trypsin used in our study is a serine protease that specifically cleaves at the carboxyl side of lysine and arginine residues). After 10 or 120 min of simulated gastric fluid digestion, more peptides that had K or R terminals were observed in the FVP–WPI group compared with the WPI group. This finding was consistent with those of previous studies [54]. This was attributed to the protection offered by FVPs, which could make the WPI keep away from gastric fluid digestion. Meanwhile, the trypsin digested samples drastically, and thus there were more peptides that had K or R terminals in the system. Abundance of peptides in the FVP–WPI group increased significantly after 120 min of digestion compared with that observed on minute 10 (Figure 6B). This was due to long-time digestion by gastric fluid. These results indicated that the formation of the FVP–WPI complexes protected WPI from digestion via pepsin, which also can increase satiety of beverages that contain FVP–WPI. On the basis of the nutrition and function of the complex, the highly stable aqueous solution produced using the complex will have broad application prospects in the future.

### 3.8. 3D Structures of Potential Binding Regions

**Figure 7:**
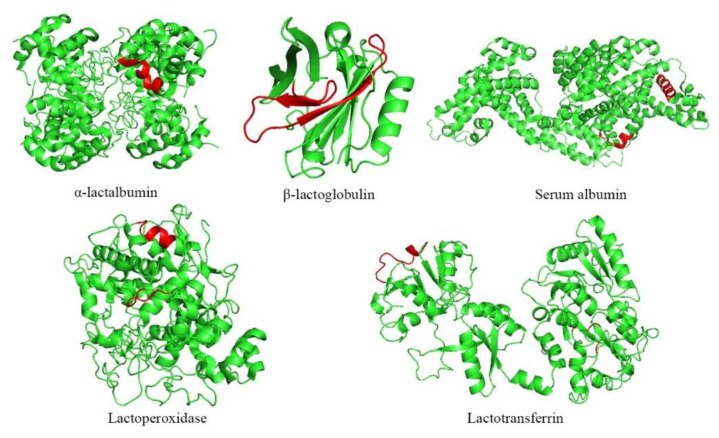
3D structure of potential binding regions in WPI (red regions indicate the potential binding regions with FVPs).

The potential binding regions were inferred by peptide abundance changes calculated via proteomics data and were mapped onto WPI sequences. Software tools such as Pymol can be used to map MS data to the protein structure to achieve data visualization [55]. The potential binding regions are shown as 3D structures in Figure 7. As shown in Figure 7, the peptide fragment “VGINYWLAHK” was probably a binding region between FVPs and alpha-lactalbumin; the peptide fragment “TKIPAVFKIDALNENK” or “IDALNENKVLVLDTDYKK” was likely docked with β-lactoglobulin. The peptides TPVSEKVTK and DAFLGSFLYEYSR were the binding region of serum albumin to FVPs. FVPs interacted with lactoperoxidase through binding with peptide LFQPTHK and DGGIDPLVR, and the peptide fragments “KANEGLTWNSLK” and “APVDAFK” were the binding region of lactotransferrin to FVPs. The previous study showed that the peptide fragment “VGINYWLAHK” was one of the binding regions of alpha-lactalbumin to *Tremella fuciformis* polysaccharide [41]. Accurate binding region of WPI and polysaccharide would be inferred through a mass of dates in further research. In this study, results about the predictions of binding regions implied that the proteomics is a useful technique that will be further developed for studying the interaction properties between polysaccharide and protein in the future.

## 4. Conclusions

The FVP–WPI complexes were prepared via noncovalent interactions of FVP and WPI. The results of our work showed that the FVP–WPI complexes dispersed well in an aqueous solution at pH 4.5, had high stability, and maintained antioxidant activities. The results of FTIR, Raman, and XRD primarily showed the differences in secondary structures and conformation between FVP–WPI and WPI. In addition, we confirmed that FVPs did form stable complexes with WPI through noncovalent bonding according to kinetics via BLI. Proteomics analysis proved that FVPs can protect WPI from gastric digestion, and inferred the binding site between FVPs and WPI, which further proved that FVPs can form stable complexes with WPI through noncovalent interactions. These results all proved that FVPs can form stable complexes with WPI at pH 4.5 and showed that FVPs are a great material to change the stability of WPI. Our results provide a foundation for the application of FVPs and WPI to develop the stability and functional properties of WPI beverages in the food industry.

## Figures and Tables

**Table 1:** The gradient conditions of the nano liquid phase system.

Composition	Time
0–8% B	0–5 min
8–32% B	5–75 min
32–90% B	75–77 min
90–0% B	82–85 min
0% B	85–90 min

**Table 2:** Content of secondary structural components of WPI and FVP–WPI.

		Structural Contribution (%)	
Structure	Frequency (cm^−1^)	WPI	FVP–WPI
α-Helix	1654	24.85 ± 0.22 ^b^	32.59 ± 0.38 ^a^
β-Sheet	1667 and 1676	46.49 ± 0.45 ^a^	31.70 ± 0.36 ^b^
β-Turn	1632 and 1684	11.29 ± 0.67 ^b^	16.08 ± 0.76 ^a^
Random coil	1643	10.87 ± 0.63 ^b^	14.85 ± 0.4 ^a^
Amino acid side	1614	4.53 ± 0.67 ^a^	4.54 ± 0.27 ^a^

Error bars indicate mean values ± standard deviations (*n* = 3), Different superscripts within a line indicate significant differences (*p* < 0.05).

## Supplementary Materials

The following are available online at https://www.mdpi.com/2304-8158/10/1/1/s1, Figure S1: Images and back scattering (BS) of FVP–WPI solutions over 60 d of storage.
