# Supplementary figures and images for: Molecular Properties of Flammulina velutipes Polysaccharide–Whey Protein Isolate (WPI) Complexes via Noncovalent Interactions
Source: Foods. 2020 Dec 22;10(1):1. doi: 10.3390/foods10010001 (PMC7821936; doi:10.3390/foods10010001)

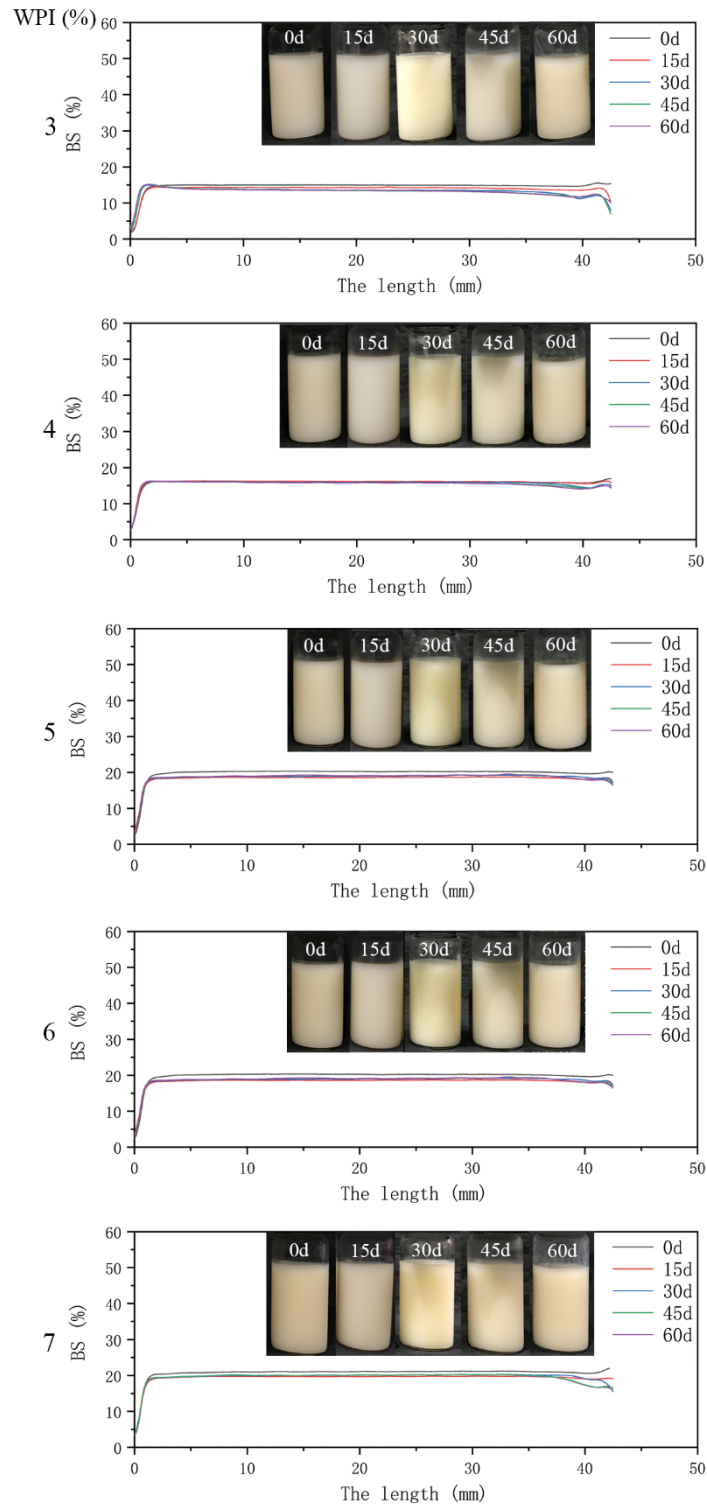

**Figure 1.** Images and Back scattering (BS) of FVP-WPI solutions over 60 d of storage.

Supplement: Supplementary file 1 [file foods-10-00001-s001.pdf]
